# A P-loop Mutation in Gα Subunits Prevents Transition to the Active State: Implications for G-protein Signaling in Fungal Pathogenesis

**DOI:** 10.1371/journal.ppat.1002553

**Published:** 2012-02-23

**Authors:** Dustin E. Bosch, Francis S. Willard, Ravikrishna Ramanujam, Adam J. Kimple, Melinda D. Willard, Naweed I. Naqvi, David P. Siderovski

**Affiliations:** 1 Department of Pharmacology, The University of North Carolina at Chapel Hill, Chapel Hill, North Carolina, United States of America; 2 Fungal Patho-Biology Group, Temasek Life Sciences Laboratory, Singapore; 3 School of Biological Sciences, Nanyang Technological University, Singapore; 4 Department of Biological Sciences, National University of Singapore, Singapore; 5 UNC Neuroscience Center and Lineberger Comprehensive Cancer Center, The University of North Carolina at Chapel Hill, Chapel Hill, North Carolina, United States of America; Purdue University, United States of America

## Abstract

Heterotrimeric G-proteins are molecular switches integral to a panoply of different physiological responses that many organisms make to environmental cues. The switch from inactive to active Gαβγ heterotrimer relies on nucleotide cycling by the Gα subunit: exchange of GTP for GDP activates Gα, whereas its intrinsic enzymatic activity catalyzes GTP hydrolysis to GDP and inorganic phosphate, thereby reverting Gα to its inactive state. In several genetic studies of filamentous fungi, such as the rice blast fungus *Magnaporthe oryzae*, a G42R mutation in the phosphate-binding loop of Gα subunits is assumed to be GTPase-deficient and thus constitutively active. Here, we demonstrate that Gα(G42R) mutants are not GTPase deficient, but rather incapable of achieving the activated conformation. Two crystal structure models suggest that Arg-42 prevents a typical switch region conformational change upon Gα_i1_(G42R) binding to GDP·AlF_4_^−^ or GTP, but rotameric flexibility at this locus allows for unperturbed GTP hydrolysis. Gα(G42R) mutants do not engage the active state-selective peptide KB-1753 nor RGS domains with high affinity, but instead favor interaction with Gβγ and GoLoco motifs in any nucleotide state. The corresponding Gα_q_(G48R) mutant is not constitutively active in cells and responds poorly to aluminum tetrafluoride activation. Comparative analyses of *M. oryzae* strains harboring either G42R or GTPase-deficient Q/L mutations in the Gα subunits MagA or MagB illustrate functional differences in environmental cue processing and intracellular signaling outcomes between these two Gα mutants, thus demonstrating the *in vivo* functional divergence of G42R and activating G-protein mutants.

## Author Summary

Heterotrimeric G-proteins function as molecular switches to convey cellular signals. When a G-protein coupled receptor encounters its ligand at the cellular membrane, it catalyzes guanine nucleotide exchange on the Gα subunit, resulting in a shift from an inactive to an active conformation. G-protein signaling pathways are conserved from mammals to plants and fungi, including the rice blast fungus *Magnaporthe oryzae*. A mutation in the Gα subunit (G42R), previously thought to eliminate its GTPase activity, leading to constitutive activation, has been utilized to investigate roles of heterotrimeric G-protein signaling pathways in multiple species of filamentous fungi. Here, we demonstrate through structural, biochemical, and cellular approaches that G42R mutants are neither GTPase deficient nor constitutively active, but rather are unable to transition to the activated conformation. A direct comparison of *M. oryzae* fungal strains harboring either G42R or truly constitutively activating mutations in two Gα subunits, MagA and MagB, revealed markedly different phenotypes. Our results suggest that activation of MagB is critical for pathogenic development of *M. oryzae* in response to hydrophobic surfaces, such as plant leaves. Furthermore, the lack of constitutive activity by Gα(G42R) mutants prompts a re-evaluation of its use in previous genetic experiments in multiple fungal species.

## Introduction

G protein-coupled receptors (GPCRs) convert extracellular signals to intracellular responses, primarily by stimulating guanine nucleotide exchange on heterotrimeric G-protein Gα subunits [1]. Upon receptor-stimulated exchange of GTP for GDP, Gα subunits undergo a conformational change, dominated by three mobile switch regions, resulting in separation of Gα from the obligate Gβγ heterodimer [2]. Switches one and two directly contact the bound guanine nucleotide and include residues critical for catalyzing GTP hydrolysis, while switch three contacts switch two in the activated conformation [3]. The nucleotide-dependent conformational shift of Gα subunits can be monitored biochemically by differential resistance to proteolysis by trypsin or altered tryptophan fluorescence spectra [4], [5]. The switch mechanism of activation is highly conserved among the mammalian Gα subunit family members, as well as in those found in fungi [6], [7]. The activated Gα and free Gβγ subunits propagate signals through numerous effectors, including adenylyl cyclases, phospholipases, ion channels, and phosphodiesterases [8]. Mammals express multiple Gα subunits which can be classified into subfamilies according to function. For example, members the Gα_i/o_ subfamily have inhibitory effects on adenylyl cylase and stimulatory effects on cGMP-phosphodiesterase, while Gα_q_ subfamily members stimulate phospholipase C isoforms, promoting hydrolysis of phosphatidylinositol bisphosphate to produce diacylglycerol and inositol triphosphate [9], [10]. Gα signaling is terminated by intrinsic hydrolysis of bound GTP to GDP, a reaction accelerated by ‘regulators of G-protein signaling’ (RGS proteins), and reversion of the Gα switch conformation to the inactive, GDP-bound state [9], [11]. Gα·GDP can then re-assemble a heterotrimer with Gβγ or, in the case of the Gα_i/o_ subfamily, engage GoLoco motif proteins that are also selective for the inactive Gα state [12]. In addition to naturally occurring conformationally selective binding partners, phage display peptides have also been engineered to discriminate between Gα·GDP and Gα·GTP. For example, the peptides KB-752 and KB-1753 selectively interact with the inactive GDP-bound and active GTP-bound states of Gα_i1_, respectively [13].

Heterotrimeric G-protein signaling components are well-characterized regulators of mammalian biology and are also utilized as sensors for extracellular cues in non-mammalian organisms, such as fungi, plants, and yeast [7], [14], [15]. The rice blast fungus, *Magnaporthe oryzae*, forms infection structures known as appressoria in response to specific environmental surface signals [16]. For example, hydrophobic, but not hydrophilic surfaces, promote appressorium formation [17]–[19]. Genetic studies have implicated a number of G-protein signaling pathway components in the regulation of *M. oryzae* pathogenesis, including a Gβ subunit (MGB1) [20], adenylyl cyclase (Mac1 or MAC) [21], cAMP phosphodiesterase (PdeH) [22], and cAMP-dependent protein kinase A (cPKA) [23]. *M. oryzae* also possesses three Gα subunits (MagA, MagB, and MagC) with sequence similarity to the Gα_s_, Gα_i_, and the fungal-specific Gα_II_ subfamilies, respectively [19], [24], [25]. Previous studies on Gα subunit deletion strains and magB mutants suggest a role for heterotrimeric G-protein signaling in *M. oryzae* growth, sexual reproduction, and appressorium formation [24], [26]. Additionally, an RGS protein (Rgs1) negatively modulates all three *M. oryzae* Gα subunits [19].

Among the most stringently conserved motifs of Gα subunits is the phosphate-binding loop (P-loop) (Figure S1). Very little variation in the P-loop sequence is seen across Gα subunits in distantly related species, including plants, fungi, and metazoans [27]. In fact, the P-loop is also conserved as a key phosphoryl group-interacting motif in ATP-binding kinases and members of the Ras GTPase superfamily [28].

A P-loop mutation to human Ras isoforms, Gly-12 to valine, is frequently found in human cancers. Ras G12V mutants are GTPase deficient, and thus constitutively active, leading to aberrant signaling and oncogenesis [29]. In fact, mutation of H-Ras Gly-12 to any residue other than proline results in constitutive activity [30]. Mutation of the corresponding P-loop residue in Gα_i1_, Gly-42 to valine, also drastically reduces its GTPase activity [31]. Structural studies of Gα_i1_(G42V) suggest that the introduced valine side chain sterically prevents appropriate positioning of Gln-204, a residue that coordinates a nucleophilic water molecule during GTP hydrolysis [31]. This glutamine is highly conserved and critical for GTPase activity; its mutation to leucine (“Q/L”) in Ras GTPases or Gα subunits also leads to constitutive activity [11], [29].

Genetic studies of heterotrimeric G-protein function in fungal species have used GTPase deficient Gα Q204L mutants (referred to as Q/L mutants). Additionally, a Gα subunit P-loop mutation, G42R, has been utilized in a similar context. Given that Gα_i1_(G42V) is GTPase-deficient and mutation of the corresponding glycine in Ras to any amino acid other than proline results in constitutive activation, it has been assumed that G42R mutants would be dominant and constitutively active [32]. Although the biochemical mechanism of the Gα G42R mutant has not previously been characterized, we and others have utilized it to probe the G-protein mediated biology of many fungal species (Table S1) [19], [26], [32]–[41].

The phosphate-binding P-loop and switch mechanism of activation are both stringently conserved among Gα subunits from mammals to fungi [6], [7] (Figure S1). For example, human RGS2 recognizes the highly similar GTP hydrolysis transition state conformations of both human Gα_q_ and a yeast Gα subunit (GPA1), such that RGS2 expression complements the deletion of an RGS protein gene in *S. cerevisiae* [42], [43]. Furthermore, chimeras of GPA1 and human Gα subunits can function in the yeast pheromone signaling pathway [44]. The residue position corresponding to Gly-42 in Gα_i1_ is within potential contact distance of residues in the switch regions of the structurally conserved Gα subfamily members [3], [10], [45]–[47]. The switch region sequences are highly conserved across mammalian Gα subfamilies, as well as in other species, including *M. oryzae, A. nidulans, and S. cerevisiae* (Figure S1). Given the sequence and structural conservation of these regions in Gα subunits, as well as the demonstrated consistent behavior of other point mutations in these regions across multiple Gα subunits (e.g. the GTPase-deficient Gα_i1_(Q204L) and the RGS-insensitive Gα_i1_(G184S) [48]), the behavior of the G42R mutation is expected to be consistent in MagA, MagB, and the mammalian Gα subunits. Since we were unable to obtain properly folded recombinant MagA or MagB proteins and no direct cellular assays of MagA or MagB activity are currently available, we utilized three mammalian Gα subunits to investigate the behavior of G42R mutants.

Here, we determine through structural, biochemical, genetic, and cellular approaches that Gα subunit G42R mutants are neither GTPase deficient nor constitutively active. Rather, the mutant arginine side chain prevents transition to the activated state upon Gα binding to GTP. Direct phenotypic analyses of *M. oryzae* strains harboring either Gα G42R mutants or the GTPase-deficient Gα Q204L suggests that a re-evaluation of previous fungal genetic data generated with the G42R mutation is required.

## Results

### The G42R mutation minimally perturbs the inactive conformation of Gα

**Figure 1:**
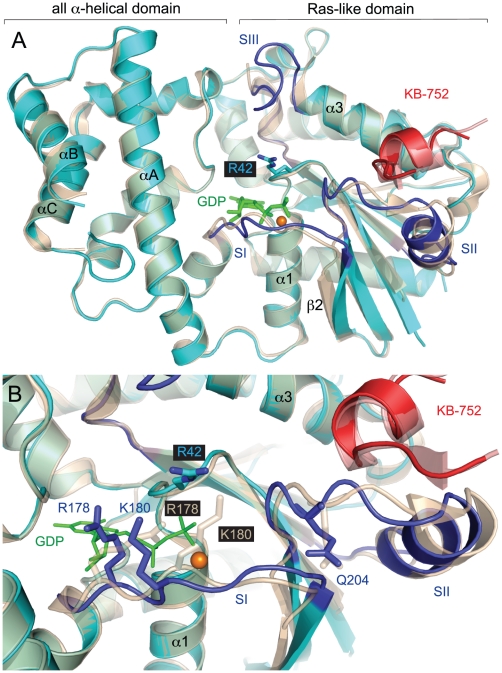
A crystal structure of Gα_i1_(G42R)·GDP in complex with the phage display peptide KB-752. (**A**) The overall structure of Gα_i1_ (cyan) with switch regions in dark blue, bound to KB-752 (red) (current study; PDB 3QE0), is overlaid on the wild type Gα_i1_·GDP/KB-752 complex (wheat/red transparency) (PDB 1Y3A). GDP is represented by green sticks and magnesium by an orange sphere. (**B**) The Arg-42 side chain extends from the P-loop, making no polar contacts with other Gα_i1_(G42R) residues, but preventing the wild type (transparent) switch conformation. Gα_i1_(G42R) residues Arg-178 and Lys-180 are displaced relative to wild type due to steric and electrostatic repulsion by Arg-42. The G42R β2 strand and switch 2 also adopt slightly different conformations. For crystallographic data collection and refinement statistics, see Table S2.

To understand how the G42R P-loop substitution affects Gα subunit structure and function, we obtained a 3.0 Å resolution crystal structure model of Gα_i1_(G42R) bound to GDP using the inactive state-selective phage display peptide KB-752 as a crystallography tool [49]. The asymmetric unit contained three Gα_i1_(G42R) subunits bound to GDP and Mg^2+^; two of three monomers were bound to the KB-752 peptide, while the third (chain C) lacked electron density for the peptide and instead displayed switch region disorder characteristic of free, GDP-bound Gα subunits [31]. For data collection and refinement statistics, see Table S1. A comparison of our model with that of wild type Gα_i1_·GDP/KB-752 (PDB id 1Y3A) revealed minor perturbations to the inactive state upon introduction of Arg-42 (Figure 1A). The side chain of Arg-42 projects away from the nucleotide-binding pocket, making no direct contacts with other Gα_i1_(G42R) residues. Switch 1 and the adjacent β2 strand adopt slightly different conformations in the mutant Gα_i1_ (Cα atoms r.m.s.d. 1.3 Å), likely because the basic residues Arg-178 and Lys-180 are electrostatically and sterically repelled from their wild type orientations by the positively charged Arg-42 side chain (Figure 1B). Arg-178 is known to stabilize the leaving phosphate group during GTP hydrolysis [11]; its perturbation in the Gα_i1_(G42R) structure model is consistent with the previously assumed GTPase deficiency of G42R mutants.

### Gα(G42R) is not GTPase deficient

**Figure 2 ppat-1002553-g002:**
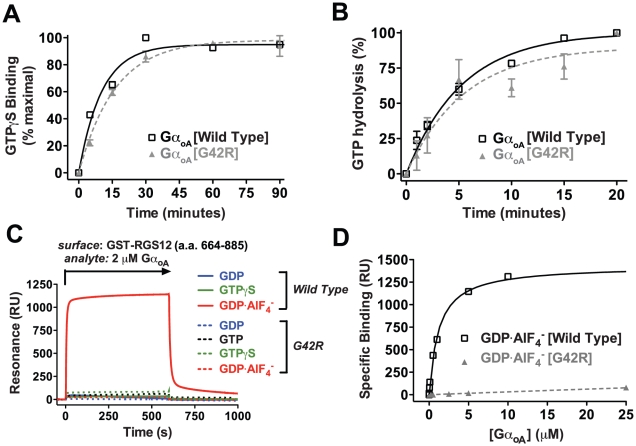
Gα_oA_(G42R) is not GTPase deficient, but retains a normal nucleotide cycle and does not interact with RGS domain. (**A**) A comparison of radiolabeled GTPγS binding by wild type Gα_oA_ (*k_on_* = 0.087±0.020 min^−1^ (s.e.m.)) and Gα_oA_(G42R) (*k_on_* = 0.062±0.010 min^−1^ (s.e.m.)) identified no significant difference in the rate of GDP release and subsequent GTP analog binding. (**B**) Gα_oA_(G42R) retained the ability to hydrolyze GTP (*k_cat_* = 0.18±0.05 min^−1^ (s.e.m.)) at a rate indistinguishable from wild type Gα_oA_ (*k_cat_* = 0.19±0.02 min^−1^ (s.e.m.)), as determined by single turnover hydrolysis assays. (**C**) Surface plasmon resonance (SPR) experiments demonstrated selective binding of the transition state-mimetic, GDP·AlF_4_^−^-bound form of Gα_oA_ to the RGS domain of RGS12. Gα_oA_(G42R) did not interact with the RGS12 RGS domain in any nucleotide state at concentrations up to 25 µM (**D**). An equilibrium binding isotherm allowed quantification of wild type Gα_oA_ affinity for RGS12 (K_D_ = 1.27±0.06 µM (s.e.m.)).

Substitution of the corresponding Gly-12 in H-Ras for any amino acid other than proline yields GTPase deficiency and constitutive activity [30]. Thus it was previously reasoned that Gα(G42R) mutants were also incapable of GTP hydrolysis [26]. Binding of GTP by purified Gα subunits can be assessed with the non-hydrolyzable GTP analog, the radionucleotide GTPγ[^35^S]. Similarly, GTPase activity can be quantified by tracking release of radioactive inorganic phosphate from [γ-^32^P]GTP-loaded Gα subunits during a single round of hydrolysis [15]. GTPγ[^35^S] radionucleotide binding and [γ-^32^P]GTP single turnover hydrolysis assays indicated that the kinetics of GTP binding and hydrolysis by the equivalent G42R mutant Gα_oA_(G42R), in the most frequent splice variant of the mammalian adenylyl cyclase inhibitory Gα_o1_, are not significantly different from wild type Gα_oA_ (Figure 2A,B). Since the nucleotide binding and hydrolysis rate of this G42R mutant was unexpectedly not perturbed, we further examined the effect of the G42R mutation on Gα interactions with known protein binding partners.

### The G42R mutation disrupts Gα interactions with RGS domains

RGS proteins accelerate the intrinsic GTPase activity of Gα subunits by stabilizing the transition state for GTP hydrolysis, a conformation mimicked by Gα binding to GDP, AlF_4_^−^, and Mg^2+^ [11]. Surface plasmon resonance (SPR) was utilized to detect optical changes upon injection of wild type or G42R mutant Gα_oA_ over a surface coated with immobilized GST-RGS12 in the presence of either GDP, GTP, the non-hydrolyzable GTP analog GTPγS, or the hydrolysis transition state-mimetic GDP·AlF_4_^−^ [50]. The RGS domain of RGS12 bound selectively to wild type Gα_oA_ in its GDP·AlF_4_^−^-bound state (K_D_ = 1.27±0.06 µM), as measured by surface plasmon resonance (SPR) [50]. However, Gα_oA_(G42R) did not engage the RGS domain in any nucleotide state at concentrations up to 25 µM (Figure 2C,D), suggesting that G42R mutants do not adopt a typical GTP hydrolysis transition state in the presence of AlF_4_^−^ and Mg^2+^ (AMF), or alternatively that Arg-42 directly interferes with RGS domain binding. A superimposition of Gα_i1_(G42R)/KB-752 and the Gα_i1_/RGS4 complex (PDB 1AGR; not shown) indicated that the mutant arginine side chain likely directly perturbs the RGS-binding surface. To further characterize nucleotide state-dependent interactions of Gα(G42R), we measured binding affinity toward three additional state-selective Gα-binding partners: Gβγ subunits, a GoLoco motif, and a phage display peptide, KB-1753 [13].

### Gα(G42R) preferentially engages inactive conformation-selective binding partners in any nucleotide state

**Figure 3 ppat-1002553-g003:**
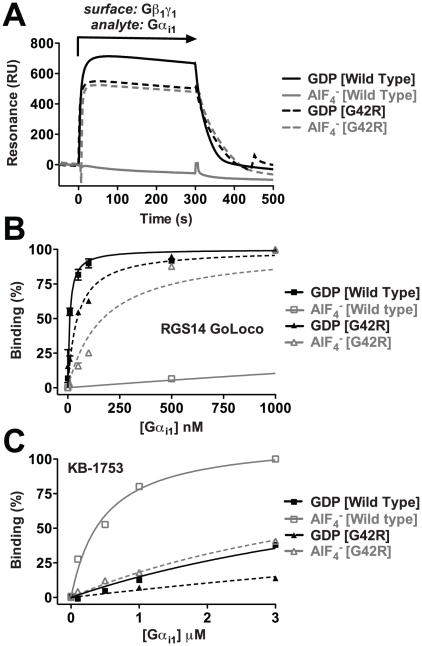
Gα_i1_(G42R) engages inactive conformation-selective binding partners in two nucleotide states. (**A**) Wild type Gα_i1_ binds Gβ_1_γ_1_ only in the GDP-bound state, as determined by SPR, while Gα_i1_(G42R) displayed no nucleotide state-selectivity of Gβ_1_γ_1_ binding when liganded with either GDP or GDP·AlF_4_^−^. (**B**) Similarly, fluorescence polarization experiments showed highly nucleotide state-selective binding of the RGS14 GoLoco motif to wild-type Gα_i1_·GDP (K_D_ = 9.0±1.1 nM (s.e.m.)) compared to the AlF_4_^−^-bound form (K_D_ = 8.7±1.0 µM (s.e.m.)), but both nucleotide states of Gα_i1_(G42R) interacted with the GoLoco motif peptide, with affinity constants of 45±7 nM (s.e.m.) and 168±27 nM (s.e.m.) for GDP and AlF_4_^−^, respectively. (**C**) The activated state-selective peptide KB-1753 preferentially bound the AlF_4_^−^-bound form of wild-type Gα_i1_ (K_D_ = 470±40 nM (s.e.m.)) compared to the GDP-bound form (K_D_ = 6.7±0.4 µM (s.e.m.)), but had low affinity for Gα_i1_(G42R) in both nucleotide states.

Gα subunits in their GDP-bound, inactive conformations form heterotrimers with Gβγ subunits [6], and the interaction is disrupted by AlF_4_^−^ or GTP binding to the Gα subunit. As expected, wild type Gα_i1_·GDP bound Gβ_1_γ_1_ as measured by SPR, but activation of the Gα subunit with GDP·AlF_4_^−^ prevented association with Gβγ (Figure 3A). However, Gα_i1_(G42R) engaged Gβ_1_γ_1_ in both nucleotide states. Interaction of Gα subunits with fluorophore-labeled peptides was assessed by detecting differences in fluorescence polarization between low molecular weight free peptide and the higher molecular weight Gα/peptide complex [51]. Similar to Gβγ, the GoLoco motif of RGS14 was highly selective for binding the GDP-bound, inactive state of wild type Gα_i1_ (K_D_ = 9.0±1.1 nM) over the activated GDP·AlF_4_^−^-bound form, as determined by fluorescence polarization (Figure 3B). Gα_i1_(G42R) displayed a much reduced selectivity for RGS14 GoLoco motif binding between the GDP and AlF_4_^−^ nucleotide states, being only 3-fold selective for the GDP form, whereas wild type Gα_i1_ is >1000-fold selective. Finally, we tested two G42R mutant nucleotide states for interaction with the active conformation-selective phage display peptide KB-1753 using fluorescence polarization [13]. As expected, KB-1753 selectively interacted with wild type Gα_i1_·GDP·AlF_4_^−^ (K_D_ = 470±40 nM) relative to GDP-bound Gα_i1_ (Figure 3C). In contrast, Gα_i1_(G42R) displayed only weak affinity for KB-1753 in either nucleotide state, as measured by fluorescence polarization. Together these data indicate that Gα(G42R) mutants preferentially engage inactive conformation-selective binding partners regardless of the bound nucleotide. To assess the conformational shift of Gα(G42R) mutants upon activation with AlF_4_^−^ or a non-hydrolyzable GTP analog, we utilized intrinsic tryptophan fluorescence and limited trypsin proteolysis.

### Gα(G42R) cannot assume the transition state-mimetic or activated conformations

**Figure 4:**
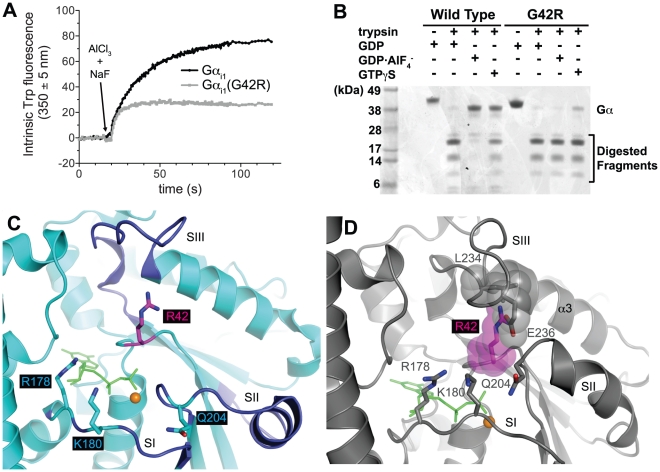
The G42R point mutation prevents Gα_i1_ from assuming the activated conformation. Upon binding GDP·AlF_4_^−^, the switch regions of Gα_i1_ undergo a conformational change, burying the switch 2 Trp-211 in a hydrophobic cleft [5]. As a result, the intrinsic tryptophan fluorescence of Gα_i1_ increases, and the activated switch conformation is protected from trypsin proteolysis, relative to the GDP-bound state. (**A**) The intrinsic tryptophan fluorescence of wild type Gα_i1_ increased upon injection of AlF_4_^−^, while the response of Gα_i1_(G42R) was blunted. (**B**) Gα_i1_ was relatively resistant to trypsin proteolysis upon loading with either GDP·AlF_4_^−^ or GTPγS. In contrast, Gα_i1_(G42R) was efficiently proteolyzed in any nucleotide state. (**C**) The Gα_i1_(G42R)·GDP/RGS14 GoLoco crystal structure model of this study (PDB 3QI2) is shown in cyan with the Arg-42 side chain in magenta sticks. GDP and magnesium are represented as green sticks and an orange sphere, respectively. The GoLoco motif peptide is excluded for clarity. For a complete model, see Figure S2. (**D**) The activated, GTPγS-bound form of wild type Gα_i1_ (PDB 1GIA) is shown in gray. Upon binding to the GTP analog, the switch regions (SI-III) of wild type Gα_i1_ converge on the phosphoryl groups of the nucleotide, resulting in a conformation recognized by effector molecules. However, the mutant Arg-42 side chain extending from the P-loop (superposed in magenta) is not sterically accommodated in a wild type-like activation state; switch 3 residues Leu-234 and Glu-236 would clash with the mutant residue. Thus, Arg-42 does not allow Gα_i1_(G42R) to assume a typical active conformation, although the critical residues Glu-204 and Arg-178 apparently can be positioned for efficient GTP hydrolysis (see Fig. 2).

Upon binding GDP·AlF_4_^−^ or GTP analogs, Gα subunits undergo conformational changes dominated by the three switch regions [52]. A tryptophan residue (Trp-211 in Gα_i1_) within switch 2 is shifted from a solvent-exposed to a buried orientation, resulting in a reduced efficiency of tryptophan fluorescence quenching that can be detected upon excitation of the Gα protein with light at 284 nm wavelength [5]. Wild-type Gα_i1_ displayed a large increase in tryptophan fluorescence upon exposure to AlF_4_^−^, indicative of a shift to the activated conformation. In contrast, the shift in tryptophan fluorescence of Gα_i1_(G42R) at the same concentration was blunted relative to wild type and occurred with faster kinetics (k_obs_ = 0.19±0.01 s^−1^ [95% C.I.], compared to k_obs_ = 0.05±0.01 s^−1^ for wild type Gα_i1_; Figure 4A).

The active and inactive states of Gα subunits are also differentially sensitive to proteolysis by trypsin; the more flexible loop conformations of Gα·GDP promote cleavage [4]. While the flexible N-terminus of wild type Gα_i1_ was cleaved in all three nucleotide states, the resulting ∼38 kDa fragment was resistant to limited trypsin proteolysis in the GDP·AlF_4_^−^ or GTP-bound conformations relative to the inactive, GDP-bound form (Figure 4B). Gα_i1_(G42R), however, was readily proteolyzed in any nucleotide state. Addition of AlF_4_^−^ had no detectable effect on Gα_i1_(G42R) resistance to trypsin proteolysis, while GTPγS provided only mild protection of the ∼38 kDa species compared to that of wild type Gα_i1_. These data further support the hypothesis that the switch regions of Gα(G42R) mutants do not assume appropriate transition state-mimetic or activated state conformations in the presence of AlF_4_^−^ and GTPγS, respectively.

### The Arg-42 side chain prevents transition of the switch regions to an active conformation

We next sought a structural explanation for the disrupted conformational switch of Gα(G42R) mutants. As previously mentioned, the Arg-42 side chain conformation, as modeled in the free GDP-bound Gα_i1_(G42R), would not allow glutamine-204 to assume its critical position for orienting the nucleophilic water required for GTP hydrolysis (Figure 1). However, unlike the G42V mutant of Gα subunits, the G42R mutant retains normal GTP hydrolysis kinetics (Figure 2). Positioning of Gln-204 for hydrolysis may be possible if the Arg-42 side chain adopts an alternate rotamer. We also crystallized Gα_i1_(G42R)·GDP in complex with the GoLoco motif from RGS14 and derived an independent structural model at 2.8 Å resolution (Table S2). In one of the two monomers of the asymmetric unit, Arg-42 adopts such an alternative rotamer that would allow Gln-204 to orient the nucleophilic water for hydrolysis (Figures 4C and S2).

Since we are presently unable to crystallize Gα_i1_(G42R) in either its GDP·AlF_4_^−^ or GTP analog-bound states, we superimposed our structural model of Gα_i1_(G42R)·GDP (excluding the RGS14 GoLoco peptide) with the previously described, wild type Gα_i1_·GTPγS (PDB id 1GIA) (Figure 4C,D). In the activated, GTPγS-bound state of wild type Gα_i1_, switches 1 and 2 converge on the nucleotide γ-phosphoryl group, while Glu-236 of switch 3 forms a new polar contact with the backbone of switch 2 [3]. The result is a convergence of the three switch regions near the P-loop to form a stable interface recognized by effector molecules. Superposition of Gα_i1_(G42R)·GDP suggests that the bulky Arg-42 side chain would not be easily accommodated by the active switch conformations observed in wild type Gα_i1_·GTPγS (Figure 4C,D). The arginine as modeled would sterically prevent the positioning of switch 3 residues Leu-234 and Glu-236 as seen in the wild type, activated state. Thus, the Arg side chain likely sterically prevents a normal activated conformation of the switch regions.

These data suggest that Arg-42 hinders attainment of the activated switch conformations seen in wild-type Gα subunits, but rotameric flexibility of the mutant side chain allows critical switch residues to effect GTP hydrolysis. Although the G42R mutants of Gα subunits have been shown to favor the inactive conformation despite retaining the ability to bind and hydrolyze GTP, we also sought to investigate their behavior in a cellular context.

### The G42R mutant is not constitutively active and displays a blunted response to stimulation by AlF_4_^−^

**Figure 5:**
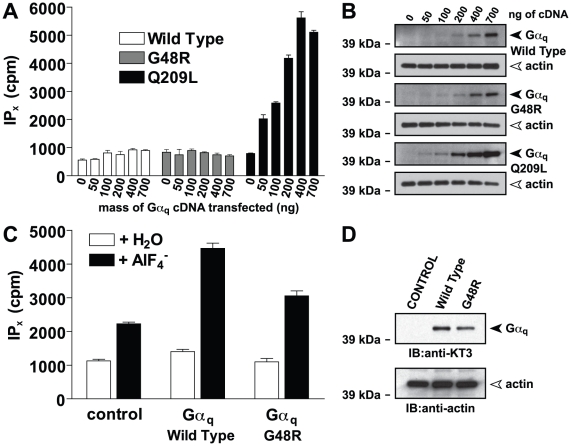
Gα_q_ G48R is not constitutively active in a cellular context. The analogous P-loop mutation in human Gα_q_, G48R, did not yield constitutive activity in contrast to the GTPase-deficient Gα_q_(Q209L) (**A,B**). Transfection of increasing amounts of Gα_q_(Q209L) markedly stimulated phospholipase C (PLC) activity in COS-7 cells, indicated by increased inositol phosphates (IP_x_) accumulation. Like wild type Gα_q_, G48R overexpression did not alter PLC activity. (**C,D**) Endogenous and overexpressed KT3 epitope-tagged wild type Gα_q_ stimulated PLC activity upon treatment with AlF_4_^−^. The response of cells expressing Gα_q_(G42R) was blunted relative to wild type Gα_q_.

To investigate the effects of G42R mutants in a signaling pathway context, we introduced the corresponding P-loop mutation into the phospholipase C stimulating mammalian Gα subunit, Gα_q_(G48R). Wild-type Gα_q_·GTP activates phospholipase Cβ (PLCβ), which in turn hydrolyzes phosphatidylinositol-4,5-bisphosphate (PIP_2_) to yield diacyl glycerol (DAG) and inositol triphosphate (IP_3_) [10]. Phospholipase C activity can be quantified by measuring accumulation of radioactive IP_3_ in cells pre-treated with tritiated inositol. Overexpression of wild type Gα_q_ in COS-7 cells had little effect on inositol phosphate accumulation, while the GTPase-deficient and constitutively active Gα_q_(Q209L) markedly stimulated PLCβ activity in a dose-dependent fashion (Figure 5A,B). Gα_q_(G48R), however, had no significant effect on PLCβ activity when overexpressed, confirming its lack of constitutive activity. Activation of PLCβ by endogenous and overexpressed Gα_q_ can be stimulated by exposure to AlF_4_^−^, since Gα_q_·GDP·AlF_4_^−^ has high affinity for PLCβ [53]. As expected, endogenous Gα_q_ was activated by AlF_4_^−^, and the effect was enhanced by overexpression of wild type Gα_q_. However, overexpressed Gα_q_(G48R) did not respond to AlF_4_^−^ stimulation to the same extent as wild type Gα_q_, reflecting its inability to assume a fully-activated conformation (Figure 5C,D).

The Gα(G42R) mutant utilized in genetic studies of fungal species, such as *Aspergillus nidulans* and the rice blast fungus *Magnaporthe oryzae*, was assumed to be GTPase deficient and thus constitutively active [26], [32], and has been used extensively to understand the biology of fungal G-protein signaling [19], [26], [32]–[41]. Since the biochemical and structural characterization of such G42R mutants (Figures 1–4 above) indicate intact GTPase activity and, instead of constitutive activity, an inability to assume the activated conformation, we sought to clarify the behavior of G42R mutations in the Gα subunits of *M. oryzae*.

### G42R and Q204L mutants of *M. oryzae* Gα subunits exhibit different effects on appressorium formation

**Figure 6:**
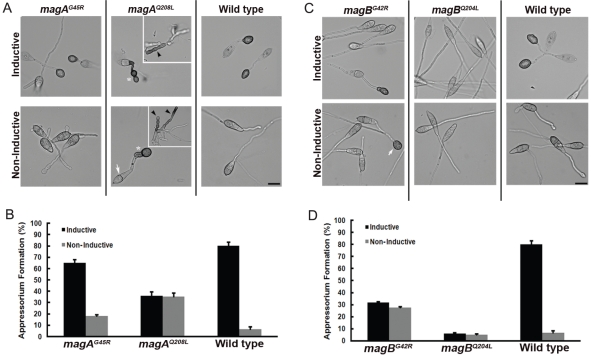
*M. oryzae* strains expressing G42R or GTPase-deficient Q204L mutant Gα subunits show disparity in appressoria formation. (**A**) Conidia harvested from the *magA^G45R^*, *magA^Q208L^* and WT strains were inoculated on inductive (plastic cover slips) or non-inductive surfaces (GelBond membrane) and assessed for the ability to form appressoria after 16 hpi (hours post inoculation). The 2-celled conidia (white arrow) of the *magA^Q208L^* produced aberrant appressorium (white asterisk) on both inductive and non-inductive surfaces. Insets represent the highly pigmented structures (black arrowhead) made by the *magA^Q208L^* strain. Scale bars = 10 µm. (**B**) Bar graph illustrating the efficiency of appressorium formation in the *magA^G45R^*, *magA^Q208L^* and wild type strains on inductive (black bar) or non-inductive surfaces (gray bar) respectively. Values represent mean ± S.E from three independent replicates involving 300 conidia per sample. (**C**) Identical experiments were conducted on the corresponding magB wild type and mutant strains. Unlike the wild type, the majority of conidia from the *magB^G42R^* strain failed to produce melanized appressoria efficiently on inductive surfaces. A small proportion of the *magB^G42R^* conidia produced mature appressoria on the non-inductive surface (indicated by the white arrow). Conidia from the *magB^Q204L^* failed to produce appressoria on both inductive and non-inductive surfaces. (**D**) Bar graph showing quantification of appressorium formation, as in (**B**).

We directly compared strains of *M. oryzae* harboring mutations in the Gα subunits MagA or MagB. Since both Gα subunits are known to regulate appressorium formation in response to inductive, hydrophobic surfaces [24], we assessed appressorium formation by GTPase-deficient Q/L and non-activatable G42R mutant strains on both hydrophobic and hydrophilic surfaces. The magA(G45R) mutant formed slightly fewer appressoria on hydrophobic, inductive surfaces than wild-type *M. oryzae*, but maintained the differential response to surface hydrophobicity (Figure 6A,B). In contrast, approximately 35% of magA(Q208L) conidia formed highly pigmented appressoria, albeit aberrant, after 16 hours, regardless of surface hydrophobicity. The magB(G42R) mutant strain resembled magA(Q208L), with ∼30% appressorium formation independent of surface hydrophobicity (Figure 6C,D). The magB(Q204L) strain, however, formed very few appressoria on either surface.

To further characterize differences between magA and magB G42R and Q/L mutant strains of *M. oryzae*, we compared colony and conidia morphology, as well as conidiation, to the wild type fungus. Both the magA and magB G42R mutants displayed different overall morphology from the corresponding Q/L mutants (Figure S3). In the case of magA(G45R), morphology was indistinguishable from the wild type. Upon exposure to light, the magA(G45R) also produced slightly fewer conidia when compared to the wild-type *M. oryzae*, but magA(Q208L) formed very few heavily pigmented, aberrant conidia (Figure 6A, inset and S4A). Both magB(G42R) and magB(Q204L) displayed enhanced conidiation relative to wild type, but those of magB(Q204L) were of a distinct morphology, with longer and thinner dimensions than either magB(G42R) or wild type (Figure S4B, C).

These data indicate that fungal Gα G42R mutants exhibit markedly different phenotypes from truly GTPase-deficient Q/L mutants, consistent with aforementioned structural, biochemical, and cellular experiments that indicate an intact GTPase activity, but a marked inability to achieve an activated conformation.

### *M. oryzae* expressing either G42R or Q204L mutant Gα subunits have differential effects on pathogenesis

**Figure 7:**
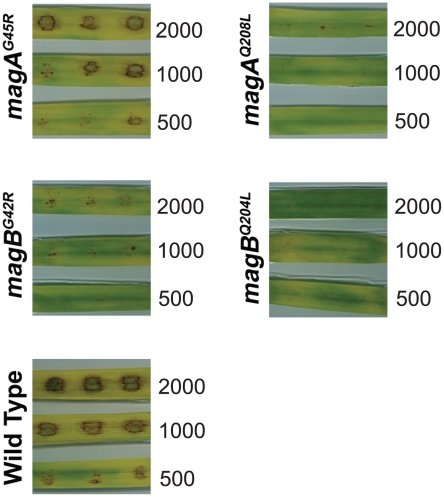
Expression of non-activatable (G42R) or GTPase-deficient (Q204L) Gα subunits differentially affects *M. oryzae* pathogenicity. Barley leaf explants were spot inoculated in triplicate with the specified number of conidia (500, 100 and 2000 per inoculation site) from the *magA^G45R^*, *magA^Q208L^*, *magB^G42R^*, *magB^Q208L^* and wild type strains and the disease symptoms scored 7d post inoculation. The *magA^G45R^* caused typical disease lesions comparable to the wild type. The *magA^Q208L^* failed to cause typical blast lesions even at high spore counts. The *magB^G42R^* caused mild disease lesions on barley leaf explants inoculated with higher concentration of spores. Under comparable conditions, conidia from the *magB^Q208L^* were incapable of causing disease.

We next determined what effect the introduction of the non-activatable G42R mutant Gα subunits has on fungal infection of barley leaves compared to constitutively active Q/L mutants. As expected, barley leaves inoculated with wild type *M. oryzae* showed the characteristic dose-dependent formation of disease lesions (Figure 7). The magA(G45R) strain showed similar pathogenicity as the wild type, consistent with intact surface-inducible appressorium formation (Figure 6B). magB(G42R) displayed a reduced ability to cause disease, although small lesions were observed at the highest inoculations tested. Both magA(Q208L) and magB(Q204L) showed drastically reduced lesion formation relative to wild type and the corresponding G42R mutants. These data indicate that constitutive activity of either MagA or MagB can suppress the ability of *M. oryzae* to penetrate and infect the plant tissue. Additionally, we conclude that the ability of MagB to achieve its activated conformation is critical for *Magnaporthe* pathogenesis.

## Discussion

Mutant Gα subunit strains have provided excellent tools for probing the functions of heterotrimeric G-proteins in many fungal species, including *Aspergillus nidulans* and *Magnaporthe oryzae* (Table S1) [19], [26], [32]–[41]. Here, we have demonstrated that the P-loop mutant, G42R, is neither GTPase deficient nor constitutively active as assumed in previous studies. Rather, Gα(G42R) is unable to undergo a typical conformational change upon binding GTP, reflected by its inability to engage RGS domains or effector-like molecules. Consistent behavior of Gα(G42R) mutations was observed in three mammalian Gα subunit family members: Gα_i1_, Gα_oA_, and Gα_q_. This finding, together with high sequence conservation surrounding the mutant residue (Figure S1) and distinct phenotypes of *M. oryzae* harboring either Gα(G42R) or truly GTPase-deficient Q/L mutants strongly support our hypothesis that MagA(G45R) and MagB(G42R) are structurally and biochemically similar to the corresponding mammalian Gα mutants. Our crystal structure models of Gα_i1_(G42R) indicates that this perturbed conformational flexibility is likely due to steric hindrance and electrostatic repulsion between the mutant Arg-42 side chain and residues of the switch regions. The preserved GTPase activity of Gα(G42R) mutants implies that Gln-204 is still able to orient a nucleophilic water during GTP hydrolysis. The structural model of Gα_i1_(G42R)·GDP bound to the GoLoco motif of RGS14 has provided a snapshot of an alternative Arg-42 rotamer that would indeed allow Gln-204 to access the orientation necessary for GTP hydrolysis. However, this rotamer still is expected to perturb the activated conformation of switch 3. We conclude that rotameric flexibility at Arg-42 allows the G42R mutant to retain GTPase activity while preventing appropriate active state switch conformations. Interestingly, previous work has identified another Gα_i1_ point mutation, K180P, that uncouples GTP hydrolysis from nucleotide-dependent conformational change [54]. Gα_i1_(K180P) is capable of hydrolyzing GTP when not in a fully activated conformation, as also seen for Gα_i1_(G42R).

Despite the retained ability of Gα(G42R) mutants to exchange and hydrolyze nucleotide, they favor an inactive state-like conformation, likely forming a less-dissociable heterotrimer with Gβγ in a cellular context, thereby reducing Gβγ/effector interactions. Since Gα(G42R) does not engage effectors with high affinity, it may be expected to behave as a dominant negative mutation; the Gα(G42R)/Gβγ heterotrimer may serve as a substrate for receptor-stimulated exchange but fail to activate downstream signaling pathways. In *Magnaporthe oryzae*, it was previously unclear why strains with magB deleted or expressing the assumedly constitutively active magB^G42R^ exhibited similar phenotypes regarding conidiation, sexual reproduction, and virulence on plant leaves [26]. The present study resolves this issue by demonstrating that the G42R mutant is not constitutively active, but likely exerts a dominant negative effect. The distinct behaviors of Gα(G42R) mutants are highlighted by a direct comparison to the truly GTPase-deficient and constitutively active Q/L mutants.

Although the magA^G45R^ and magB^G42R^ mutant strains do not reflect constitutive Gα subunit activity, as previously assumed [26], [32], they do provide insight into fungal pathogenic development. A phenotypic deficiency upon expression of a Gα(G42R) mutant suggests that specific activation of the Gα of interest and subsequent engagement of its downstream effectors is necessary for a particular function of a cell or organism. For instance, both magB deletion [24] and magB^G42R^ mutant strains display drastically reduced induction of appressoria by hydrophobic surfaces, while magA deletion [24] and magA^G45R^ mutations each have minimal effects. Thus, it is likely that MagB transduces an external surface hydrophobicity signal, presumably through a GPCR. Use of the magB^G42R^ mutant suggests that the conformational change accompanying MagB activation is necessary for the selective development of appressoria on hydrophobic surfaces (Figure S6). It remains to be determined whether the Gα or Gβγ subunits or both propagate signals required for appressorium formation and disease lesion formation in *M. oryzae*. Direct evidence of interactions between *Magnaporthe* heterotrimeric G-protein subunits and effector molecules is currently lacking. However, phenotypic similarities between the Gα subunit mutant and deletion strains [20], [24], [26], Gβ subunit (MGB1) deletion [20], adenylyl cylase (Mac1) deletion [21], and cAMP phosphodiesterase (PdeH) deletion [22], suggest that MagA and MagB may modulate cellular cAMP level through mechanisms similar to those of mammalian Gα_s_ and Gα_i/o_.

In conclusion, Gα(G42R) mutants are incapable of assuming a typical activated conformation, but their retained ability to hydrolyze GTP indicates an uncoupling of conformational change and enzymatic activity. Since G42R mutants are unable to separate from Gβγ or to activate effectors, they provide tools for dissecting the functions of Gα subunits in cellular contexts. Utilizing both G42R and constitutively active Q/L mutants of two Gα subunits, we postulate a critical role for MagB activation in response to growth on hydrophobic surfaces, leading to appressorium formation in the rice blast fungus, *M. oryzae*.

## Materials and Methods

### Chemicals and other assay materials

Unless otherwise noted, all chemicals were the highest grade available from Sigma or Fisher Scientific. Peptides were synthesized by Fmoc (*N*-(9-fluorenyl)methoxycarbonyl) group protection, purified by HPLC, and confirmed using mass spectrometry by the Tufts University Core Facility (Medford, MA). Peptides used for crystallography and biophysical studies have been previously reported: FITC-RGS14 GoLoco [55], RGS14 GoLoco [56], FITC-KB-1753 [13], and KB-752 [49].

### Protein purification

Although we were unable to obtain properly folded, purified *M. oryzae* Gα subunits, the P-loop and surrounding switch regions are highly conserved from mammals to fungi (Figures S1). Thus, we utilized the readily available purified Gα_i1_ and Gα_oA_ and corresponding G42R mutants. For biochemical experiments, full-length, hexahistidine-tagged Gα_i1_ and Gα_oA_, and G42R mutants thereof, were purified from *E. coli* by NTA affinity and gel filtration chromatography as previously described [57] (see Figure S5). A GST fusion of the RGS12 RGS domain (aa 664–885) was purified as described [58]. Biotinylated Gβ_1_γ_1_ was purified as described [59]. For crystallization, an N-terminally truncated (ΔN30) Gα_i1_(G42R) was expressed and purified by NTA affinity chromatography; the hexahistidine tag was cleaved by TEV protease, and the Gα subunit further purified by ion exchange (SourceQ, GE Healthcare) and gel filtration chromatography. Purified Gα_i1_(G42R) was loaded with excess GppNHp or GDP for 3 hours at room temperature and concentrated to 15 mg/mL in GppNHp crystallization buffer (50 mM HEPES pH 8.0, 10 mM MgCl_2_, 10 µM GppNHp, 1 mM EDTA, 5 mM DTT) or GDP crystallization buffer (10 mM Tris pH 7.5, 1 mM MgCl_2_, 5% v/v glycerol, 5 mM DTT).

### Crystallization and structure determination

The complex of Gα_i1_(G42R) and synthetic KB-752 peptide was obtained by mixing a 1∶1.5 molar ratio of protein to peptide in GppNHp crystallization buffer. Despite loading of Gα_i1_(G42R) and crystallization in the presence of GppNHp, the crystal lattice contained Gα_i1_(G42R) liganded with GDP and bound to KB-752. The selectivity of KB-752 for the GDP bound state [49] may account for the apparent absence of GppNHp. Crystals of Gα_i1_(G42R)·GDP/KB-752 were obtained by vapor diffusion from hanging drops containing a 1∶1 (v/v) ratio of protein/peptide solution to well solution (17% (w/v) PEG MME 5000, 200 mM MgCl_2_, 100 mM HEPES pH 7.0). Hexagonal rod crystals (∼300×100×100 µm) formed in 5 days at 18°C exhibited the symmetry of space group P6_1_22 (*a* = *b* = 106.6, *c* = 455.1, and α = β = 90°, γ = 120°) and contained two Gα_i1_(G42R)·GDP/KB-752 dimers and one Gα_i1_(G42R)·GDP monomer in the asymmetric unit. For data collection at 100K, crystals were serially transferred into well solution supplemented with 30% saturated sucrose in 10% increments for ∼30 s, followed by plunging into liquid nitrogen. A native data set was collected at the SER-CAT 22-ID beamline at the Advanced Photon Source (Argonne National Laboratory). Data were processed using the HKL-2000 program [60]. The crystal structure of the wild type Gα_i1_/KB-752 heterodimer (PDB 1Y3A [49]), excluding the KB-752 peptide, nucleotide, and waters was used as a search model for molecular replacement using the Phaser program [61]. Refinement was carried out using phenix.refine [62], consisting of conjugate gradient minimization and refinement of individual atomic displacement and translation-libration-screw parameters, interspersed with manual revisions of the model using the Coot program [63]. For data collection and refinement statistics and a list of residues that could not be located in the electron density, see Table S2.

The complex of Gα_i1_(G42R) and the RGS14 GoLoco motif peptide was generated by mixing a 1∶1.5 molar ratio of protein to peptide in GDP crystallization buffer. Crystals of the complex were obtained by vapor diffusion from hanging drops containing a 1∶1 ratio of protein/peptide solution to well solution (1.7 M ammonium sulfate, 100 mM sodium acetate pH 5.0, 200 mM MgCl_2_, 10% (w/v) glycerol). Crystals (∼200×200×50 µm) formed in 2–5 days at 18°C and exhibited the symmetry of space group C222_1_ (*a* = 70.0, *b* = 131.0, *c* = 203.3, and α = β = γ = 90°) and contained two Gα_i1_(G42R)/GoLoco motif heterodimers in the asymmetric unit. Diffraction data were collected and processed, and the model refined as described for Gα_i1_(G42R)/KB-752, above. The crystal structure of Gα_i1_(Q147L)/RGS14 GoLoco motif (PDB 2OM2 [51]), excluding the peptide, nucleotide and waters was used as a molecular replacement search model. All structural images were made with PyMOL (Schrödinger LLC, Portland, OR).

### Nucleotide binding and hydrolysis assays

The [^35^S]GTPγS filter-binding assay used to measure rates of spontaneous GDP release from wild type and mutant Gα_oA_ was conducted as described previously [64]. Intrinsic GTP hydrolysis rates of Gα_oA_ and mutants were assessed by monitoring ^32^P-labeled inorganic phosphate production during a single round of GTP hydrolysis as described previously [65].

### Surface plasmon resonance assays

Optical detection of protein/protein interactions by surface plasmon resonance was performed using a Biacore 3000 (GE Healthcare). Carboxymethylated dextran (CM5) sensor chips (GE Healthcare) with covalently bound anti-GST antibody surfaces were created as described previously [50]. The GST-RGS12 RGS domain protein and GST alone (serving as a negative control) were separately immobilized on SPR chip surfaces. Biotinylated Gβ_1_γ_1_ and mNOTCH peptide (serving as a negative control) were separately immobilized on a streptavidin (SA) sensor chip (GE Healthcare) as described previously [50].

### Fluorescence polarization measurements

All polarization experiments were conducted using a PHERAstar microplate reader (BMG Labtech, Offenburg, Germany), essentially as described previously [51].

### Intrinsic tryptophan fluorescence measurements of Gα activation

Changes in tryptophan fluorescence of Gα_i1_ subunits were measured to assess activation by GDP·AlF_4_^−^, as described previously [51]. Activation of Gα subunits results in translocation of a conserved switch 2 tryptophan into a hydrophobic pocket, increasing the quantum yield of tryptophan fluorescence [5]. Fluorescence intensity traces shown are representative of triplicate experiments.

### Limited trypsin proteolysis

Gα subunits are relatively protected from trypsin-mediated proteolysis in the GDP·AlF_4_^−^ and GTP analog-bound, activated states [4]. Ten µg of wild type or mutant Gα_i1_ in 50 mM HEPES (pH 8.0), 1 mM EDTA, 5 mM DTT, 0.05% C_12_E_10_, and 10 mM MgCl_2_ were incubated for three hours at room temperature with either 100 µM GDP, 100 µM GTPγS, or 100 µM GDP, 20 mM NaF, and 60 µM AlCl_3_. Five hundred ng of *N*-Tosyl-L-phenylalanine chloromethyl ketone (TPCK)-treated trypsin was added to each reaction, followed by a 10-minute incubation at room temperature. Proteolysis was stopped by addition of SDS-PAGE sample buffer and boiling. Samples were subjected to SDS-PAGE and stained with Coomassie Blue.

### Quantitation of phospholipase C (PLC) activity

COS-7 cells in 12-well culture dishes were transfected with KT3-tagged wild type or mutant Gα_q_, metabolically labeled with 1 µCi of [^3^H]inositol/well and assayed for inositol phosphate accumulation using Dowex chromatography as described previously [66]. For AlF_4_^−^ stimulation experiments, final concentrations of 10 mM NaF and 30 µM AlCl_3_ were added to cell media. To determine wild type and mutant Gα_q_ expression levels, cells were lysed in SDS-PAGE sample buffer. Proteins separated by electrophoresis were immunoblotted with anti-KT3 antibody (Covance) or anti-actin antibody (Sigma).

### Fungal strains, growth, and culture conditions

The *M. oryzae* wild-type strain B157 was obtained from the Directorate of Rice Research (Hyderabad, India). *Magnaporthe* strains carrying individual point mutations in the Gα subunits, namely: *magA^G45R^*, *magA^Q208L^*, *magB^G42R^*, *magB^Q208L^* have been described previously together with the *rgs1*Δ mutant [19]. Wild type and mutant strains cultures were maintained at 28°C in the dark on Prune Agar medium plates (PA; per L: 40 mL prune juice, 5 g lactose, 5 g Sucrose, 1 g yeast extract and 20 g agar, pH 6.5). Assessment of the radial growth, aerial hyphae and colony characteristics was carried out as previously described [22]. Conidiation was induced in the *Magnaporthe* colonies through exposure to continuous incandescent light at room temperature for 6 days.

### Evaluation of conidiation status

Conidia were harvested by scraping the surface growth in water with an inoculation loop. The suspension was filtered through two layers of Miracloth (Calbiochem, San Diego, USA), collected in Falcon tubes (BD Biosciences, USA), vortexed for a minute to ensure complete detachment of conidia from the mycelia, and then pelleted by centrifugation at 3,000 rpm for 15 minutes. The conidia were washed twice and re-suspended in a fixed volume of sterile water. Prior to harvesting the spores, the radius of each colony was measured to calculate the surface area of the colony. Conidia produced by a given colony were quantified using a hemocytometer and reported as the total number of conidia present per unit area of the colony.

### Appressoria formation assays

Droplets (20 µl containing 500 conidia) of conidial suspension were placed on plastic cover slips (hydrophobic surface) or hydrophilic side of GelBond membrane (Lonza Walkersville Inc., USA) and incubated in a humid chamber at room temperature. The total number of appressoria formed by each strain on either surface was quantified at 16 hpi (hours post inoculation).

### Evaluation of pathogenicity in *Magnaporthe* strains

For pathogenicity assays, leaves from two week old barley seedlings were cut into smaller pieces (2–3 cm long) and washed in sterile water, following which the leaf bits were rinsed for 45 seconds in 40% ethanol. The leaf pieces were then washed twice with sterile antibiotic-containing distilled water. The washed leaves were placed on kinetin agar plates (2 mg/mL kinetin, 1% agar). Conidia were quantified and a dilution series of the conidial suspension was inoculated on detached barley leaves at the required concentrations. The samples were incubated in a humidified growth chamber with a 16 h light/8 h dark cycle at 22°C. Disease symptoms were assessed 5–7 days post inoculation.

### Microscopic analysis

Samples were observed on a BX51 (Olympus, Japan) microscope equipped with UPlan FL N 60X/1.25 Oil objective with appropriate filter sets. Bright field images were captured using a Cool SNAP HQ camera (Photometrics, USA) and processed using Image J (National Institutes of Health, USA), MetaVue (Universal Imaging, USA) and Adobe Photoshop 7.0 (Adobe Inc, USA).

## Supporting Information

Figure S1 **The Gα subunit P-loop is highly conserved in fungi and mammals.** The β1 strands, α1 helices, and intervening P-loops (gray), as well as the three switch regions of selected Gα subunits from humans and fungi are aligned. Nucleotide contacting residues are highlighted by black circles, and the mutated glycine by an arrowhead.
(EPS)

Figure S2 **Arg-42 adopts an alternate rotamer in the crystal structure model of Gα_i1_(G42R)·GDP/RGS14 GoLoco motif.** Gα_i1_(G42R) is shown in cyan with switch regions in dark blue and selected side chains in sticks. GDP is represented as green sticks, and a portion of the RGS14 GoLoco motif is orange. GoLoco motif residues 511 and 512 were disordered in the crystal structure; the cartoon shown is truncated at residue 510 (PDB 3QI2). The side chain of Arg-42 adopts a different rotamer than that seen in Gα_i1_(G42R)·GDP/KB-752 (magenta sticks). Instead, the Arg side chain forms direct polar contacts with Glu-245 of Gα_i1_(G42R) and the backbone carbonyl group of Val-507 from the RGS14 GoLoco motif. Arg-42 also coordinates a well-ordered water molecule (yellow sphere) with Arg-242 and Gln-147 of Gα_i1_(G42R). This Arg-42 rotamer would sterically prevent switch 3 from approaching the nucleotide upon binding to GTP. However, there is room for Arg-178 and Gln-204 to potentially assume their critical positions for GTP hydrolysis, providing a possible rationale for the normal GTPase activity of Gα_i1_(G42R).
(EPS)

Figure S3 ***M. oryzae*** **colony and growth characteristics.** Morphology of the *magA^G45R^*, *magA^Q208L^*, *magB^G42R^*, *magB^Q208L^*, WT (wild-type) and *rgs1*Δ colonies. The indicated strains were grown in the dark on prune agar medium for a week and photographed (upper panels). The *magB^Q208L^* mutation lead to reduced rate radial growth. The radius of the *magB^Q208L^* colony was 2.24±0.03 cm compared to 2.52±0.03 cm in the *magB^G42R^* or the WT strain, when grown under identical conditions for a period of seven days at 28°C in the dark. Values represent the mean ± SE (n = 5 colonies per strain; p<0.001). The lower panels represent cross sections at near-median planes. The *magA^Q208L^* showed dramatic reduction in aerial hyphal growth, compared to the *magA^G45R^* and WT. The *magB^G42R^* and *magB^Q208L^* mutants showed reduced aerial hyphal growth compared to the WT strain.
(EPS)

Figure S4 ***M. oryzae*** **conidiation defects and conidial morphology.** Comparative quantitative analysis of conidiation in the *magA^G45R^*, *magA^Q208L^*, *magB^G42R^ magB^Q208L^* and wild type strains. The indicated strains were initially grown in the dark for a day and then exposed to constant illumination for 6 days. Data represents mean ± SE based on three independent replicates. (**A**) Conidia per surface area unit were quantified for all five strains. Both *magA^G45R^* and *magA^Q208L^* produced fewer conidia than wild type fungi, although *magA^Q208L^* produced statistically significantly few conidia than *magA^G45R^*. The asterisk indicates the heavily pigmented aberrant structures and conidia with a single septum produced predominantly by the *magA^Q208L^* mutant. *magB^G42R^* and *magB^Q208L^* both displayed an increased number of conidia compared to wild type. (**B**) Conidia from *magB^Q208L^* displayed a thin, elongated morphology, while those of *magB^G42R^* were similar to wild type. (**C**) The dimensions (length and width) of conidia from the indicated strains were quantified. Values represent the mean ± SE (n = 200 conidia per strain).
(EPS)

Figure S5 **Purification of Gα_i1_ and Gα_o_ G42R mutants.** Wild type and G42R Gα_i1_ and Gα_o_ were purified from *E. coli* by affinity chromatography, separated by SDS PAGE, and stained with Coomassie blue.
(EPS)

Figure S6 **Activation of the Gα subunit MagB is required for selective appressorium formation on hydrophobic surfaces.** Based on genetic data from the present and previous studies, a model of MagB-mediated regulation of appressorium formation in *M. oryzae* is hypothesized. Rgs1 was previously shown to modulate appressorium formation by negatively regulating MagA and MagB [19]. Experiments involving G42R and Q/L mutants of Gα subunits, from the present study, implicate MagB activation as a vital component of surface hydrophobicity sensing, putatively through a heptahelical GPCR.
(EPS)

Table S1 **Previous studies utilizing G42R mutations in fungal Gα subunits.** Investigations into Gα subunit function in multiple species have included G42R point mutations. In each case, the G42R mutant was assumed to be GTPase-deficient and constitutively active.
(PDF)

Table S2 **Data collection and refinement statistics for Gα_i1_(G42R) complexes.**
(PDF)

## References

[1] Rosenbaum DM, Rasmussen SG, Kobilka BK (2009). The structure and function of G-protein-coupled receptors.. Nature.

[2] Wall MA, Coleman DE, Lee E, Iniguez-Lluhi JA, Posner BA (1995). The structure of the G protein heterotrimer Gi alpha 1 beta 1 gamma 2.. Cell.

[3] Coleman DE, Berghuis AM, Lee E, Linder ME, Gilman AG (1994). Structures of active conformations of Gi alpha 1 and the mechanism of GTP hydrolysis.. Science.

[4] Mazzoni MR, Hamm HE (1996). Interaction of transducin with light-activated rhodopsin protects it from proteolytic digestion by trypsin.. J Biol Chem.

[5] Higashijima T, Ferguson KM, Sternweis PC, Ross EM, Smigel MD (1987). The effect of activating ligands on the intrinsic fluorescence of guanine nucleotide-binding regulatory proteins.. J Biol Chem.

[6] Oldham WM, Hamm HE (2008). Heterotrimeric G protein activation by G-protein-coupled receptors.. Nat Rev Mol Cell Biol.

[7] Dohlman HG, Slessareva JE (2006). Pheromone signaling pathways in yeast.. Sci STKE.

[8] Tesmer JJ, Sprang SR (1998). The structure, catalytic mechanism and regulation of adenylyl cyclase.. Curr Opin Struct Biol.

[9] Siderovski DP, Willard FS (2005). The GAPs, GEFs, and GDIs of heterotrimeric G-protein alpha subunits.. Int J Biol Sci.

[10] Waldo GL, Ricks TK, Hicks SN, Cheever ML, Kawano T (2010). Kinetic scaffolding mediated by a phospholipase C-beta and G_q_ signaling complex.. Science.

[11] Berman DM, Wilkie TM, Gilman AG (1996). GAIP and RGS4 are GTPase-activating proteins for the G_i_ subfamily of G protein alpha subunits.. Cell.

[12] Willard FS, Kimple RJ, Siderovski DP (2004). Return of the GDI: the GoLoco motif in cell division.. Annu Rev Biochem.

[13] Johnston CA, Willard FS, Ramer JK, Blaesius R, Roques CN (2008). State-selective binding peptides for heterotrimeric G-protein subunits: novel tools for investigating G-protein signaling dynamics.. Comb Chem High Throughput Screen.

[14] Li L, Wright SJ, Krystofova S, Park G, Borkovich KA (2007). Heterotrimeric G protein signaling in filamentous fungi.. Annu Rev Microbiol.

[15] Johnston CA, Willard MD, Kimple AJ, Siderovski DP, Willard FS (2008). A sweet cycle for *Arabidopsis* G-proteins: Recent discoveries and controversies in plant G-protein signal transduction.. Plant Signal Behav.

[16] Lee YH, Dean RA (1993). cAMP Regulates Infection Structure Formation in the Plant Pathogenic Fungus *Magnaporthe grisea*.. Plant Cell.

[17] Talbot NJ, Ebbole DJ, Hamer JE (1993). Identification and characterization of *MPG1*, a gene involved in pathogenicity from the rice blast fungus *Magnaporthe grisea*.. Plant Cell.

[18] Beckerman JL, Ebbole DJ (1996). *MPG1*, a gene encoding a fungal hydrophobin of *Magnaporthe grisea*, is involved in surface recognition.. Mol Plant Microbe Interact.

[19] Liu H, Suresh A, Willard FS, Siderovski DP, Lu S (2007). Rgs1 regulates multiple Galpha subunits in *Magnaporthe* pathogenesis, asexual growth and thigmotropism.. EMBO J.

[20] Nishimura M, Park G, Xu JR (2003). The G-beta subunit MGB1 is involved in regulating multiple steps of infection-related morphogenesis in *Magnaporthe grisea*.. Mol Microbiol.

[21] Choi W, Dean RA (1997). The adenylate cyclase gene *MAC1* of *Magnaporthe grisea* controls appressorium formation and other aspects of growth and development.. Plant Cell.

[22] Ramanujam R, Naqvi NI (2010). PdeH, a high-affinity cAMP phosphodiesterase, is a key regulator of asexual and pathogenic differentiation in *Magnaporthe oryzae*.. PLoS Pathog.

[23] Adachi K, Hamer JE (1998). Divergent cAMP signaling pathways regulate growth and pathogenesis in the rice blast fungus *Magnaporthe grisea*.. Plant Cell.

[24] Liu S, Dean RA (1997). G protein alpha subunit genes control growth, development, and pathogenicity of *Magnaporthe grisea*.. Mol Plant Microbe Interact.

[25] Bolker M (1998). Sex and crime: heterotrimeric G proteins in fungal mating and pathogenesis.. Fungal Genet Biol.

[26] Fang EG, Dean RA (2000). Site-directed mutagenesis of the *magB* gene affects growth and development in *Magnaporthe grisea*.. Mol Plant Microbe Interact.

[27] Temple BR, Jones CD, Jones AM (2010). Evolution of a signaling nexus constrained by protein interfaces and conformational states.. PLoS Comput Biol.

[28] Saraste M, Sibbald PR, Wittinghofer A (1990). The P-loop–a common motif in ATP- and GTP-binding proteins.. Trends Biochem Sci.

[29] Campbell PM, Der CJ (2004). Oncogenic Ras and its role in tumor cell invasion and metastasis.. Semin Cancer Biol.

[30] Seeburg PH, Colby WW, Capon DJ, Goeddel DV, Levinson AD (1984). Biological properties of human c-Ha-ras1 genes mutated at codon 12.. Nature.

[31] Raw AS, Coleman DE, Gilman AG, Sprang SR (1997). Structural and biochemical characterization of the GTPgammaS-, GDP.P_i_-, and GDP-bound forms of a GTPase-deficient Gly42→Val mutant of Gialpha1.. Biochemistry.

[32] Yu JH, Wieser J, Adams TH (1996). The *Aspergillus* FlbA RGS domain protein antagonizes G protein signaling to block proliferation and allow development.. EMBO J.

[33] Hicks JK, Yu JH, Keller NP, Adams TH (1997). *Aspergillus* sporulation and mycotoxin production both require inactivation of the FadA G alpha protein-dependent signaling pathway.. EMBO J.

[34] Shimizu K, Keller NP (2001). Genetic involvement of a cAMP-dependent protein kinase in a G protein signaling pathway regulating morphological and chemical transitions in *Aspergillus nidulans*.. Genetics.

[35] Zuber S, Hynes MJ, Andrianopoulos A (2002). G-protein signaling mediates asexual development at 25 degrees C but has no effect on yeast-like growth at 37 degrees C in the dimorphic fungus *Penicillium mameffei*.. Eukaryot Cell.

[36] Zuber S, Hynes MJ, Andrianopoulos A (2003). The G-protein alpha-subunit GasC plays a major role in germination in the dimorphic fungus *Penicillium marneffei*.. Genetics.

[37] Garcia-Rico RO, Martin JF, Fierro F (2007). The *pga1* gene of *Penicillium chrysogenum* NRRL 1951 encodes a heterotrimeric G protein alpha subunit that controls growth and development.. Res Microbiol.

[38] Han KH, Kim JH, Moon H, Kim S, Lee SS (2008). The *Aspergillus nidulans esdC* (early sexual development) gene is necessary for sexual development and is controlled by *veA* and a heterotrimeric G protein.. Fungal Genet Biol.

[39] Garcia-Rico RO, Fierro F, Mauriz E, Gomez A, Fernandez-Bodega MA (2008). The heterotrimeric Galpha protein *pga1* regulates biosynthesis of penicillin, chrysogenin and roquefortine in *Penicillium chrysogenum*.. Microbiology.

[40] Garcia-Rico RO, Fierro F, Martin JF (2008). Heterotrimeric Galpha protein Pga1 of *Penicillium chrysogenum* controls conidiation mainly by a cAMP-independent mechanism.. Biochem Cell Biol.

[41] Garcia-Rico RO, Martin JF, Fierro F (2011). Heterotrimeric Galpha protein Pga1 from *Penicillium chrysogenum* triggers germination in response to carbon sources and affects negatively resistance to different stress conditions.. Fungal Genet Biol.

[42] Siderovski DP, Hessel A, Chung S, Mak TW, Tyers M (1996). A new family of regulators of G-protein-coupled receptors?. Curr Biol.

[43] Kimple AJ, Soundararajan M, Hutsell SQ, Roos AK, Urban DJ (2009). Structural determinants of G-protein alpha subunit selectivity by regulator of G-protein signaling 2 (RGS2).. J Biol Chem.

[44] Takesono A, Cismowski MJ, Ribas C, Bernard M, Chung P (1999). Receptor-independent activators of heterotrimeric G-protein signaling pathways.. J Biol Chem.

[45] Lambright DG, Noel JP, Hamm HE, Sigler PB (1994). Structural determinants for activation of the alpha-subunit of a heterotrimeric G protein.. Nature.

[46] Slep KC, Kercher MA, Wieland T, Chen CK, Simon MI (2008). Molecular architecture of Galpha_o_ and the structural basis for RGS16-mediated deactivation.. Proc Natl Acad Sci U S A.

[47] Tesmer JJ, Sunahara RK, Gilman AG, Sprang SR (1997). Crystal structure of the catalytic domains of adenylyl cyclase in a complex with G_s_alpha·GTPgammaS.. Science.

[48] Kaur K, Kehrl JM, Charbeneau RA, Neubig RR (2011). RGS-insensitive Galpha subunits: probes of Galpha subtype-selective signaling and physiological functions of RGS proteins.. Methods Mol Biol.

[49] Johnston CA, Willard FS, Jezyk MR, Fredericks Z, Bodor ET (2005). Structure of Galpha(i1) bound to a GDP-selective peptide provides insight into guanine nucleotide exchange.. Structure.

[50] Hutsell SQ, Kimple RJ, Siderovski DP, Willard FS, Kimple AJ (2010). High-affinity immobilization of proteins using biotin- and GST-based coupling strategies.. Methods Mol Biol.

[51] Bosch DE, Kimple AJ, Sammond DW, Muller RE, Miley MJ (2010). Structural determinants of affinity enhancement between GoLoco motifs and G-protein alpha subunit mutants.. J Biol Chem.

[52] Wall MA, Posner BA, Sprang SR (1998). Structural basis of activity and subunit recognition in G protein heterotrimers.. Structure.

[53] Smrcka AV, Hepler JR, Brown KO, Sternweis PC (1991). Regulation of polyphosphoinositide-specific phospholipase C activity by purified G_q_.. Science.

[54] Thomas CJ, Du X, Li P, Wang Y, Ross EM (2004). Uncoupling conformational change from GTP hydrolysis in a heterotrimeric G protein alpha-subunit.. Proc Natl Acad Sci U S A.

[55] Willard FS, Zheng Z, Guo J, Digby GJ, Kimple AJ (2008). A point mutation to Galpha_i_ selectively blocks GoLoco motif binding: direct evidence for Galpha·GoLoco complexes in mitotic spindle dynamics.. J Biol Chem.

[56] Kimple RJ, Kimple ME, Betts L, Sondek J, Siderovski DP (2002). Structural determinants for GoLoco-induced inhibition of nucleotide release by Galpha subunits.. Nature.

[57] Kimple AJ, Yasgar A, Hughes M, Jadhav A, Willard FS (2008). A high throughput fluorescence polarization assay for inhibitors of the GoLoco motif/G-alpha interaction.. Comb Chem High Throughput Screen.

[58] Willard FS, Kimple AJ, Johnston CA, Siderovski DP (2005). A direct fluorescence-based assay for RGS domain GTPase accelerating activity.. Anal Biochem.

[59] Johnston CA, Afshar K, Snyder JT, Tall GG, Gonczy P (2008). Structural determinants underlying the temperature-sensitive nature of a Galpha mutant in asymmetric cell division of *Caenorhabditis elegans*.. J Biol Chem.

[60] Otwinowski Z, Minor W (1997). Processing of X-ray Diffraction Data Collected in Oscillation Mode.. Methods Enzymol.

[61] McCoy AJ, Grosse-Kunstleve RW, Adams PD, Winn MD, Storoni LC (2007). Phaser crystallographic software.. J Appl Crystallogr.

[62] Adams PD, Afonine PV, Bunkoczi G, Chen VB, Davis IW (2010). PHENIX: a comprehensive Python-based system for macromolecular structure solution.. Acta Crystallogr D Biol Crystallogr.

[63] Emsley P, Lohkamp B, Scott WG, Cowtan K (2010). Features and development of Coot.. Acta Crystallogr D Biol Crystallogr.

[64] Afshar K, Willard FS, Colombo K, Johnston CA, McCudden CR (2004). RIC-8 is required for GPR-1/2-dependent Galpha function during asymmetric division of *C. elegans* embryos.. Cell.

[65] Johnston CA, Lobanova ES, Shavkunov AS, Low J, Ramer JK (2006). Minimal determinants for binding activated G alpha from the structure of a G alpha(i1)-peptide dimer.. Biochemistry.

[66] Wing MR, Houston D, Kelley GG, Der CJ, Siderovski DP (2001). Activation of phospholipase C-epsilon by heterotrimeric G protein betagamma-subunits.. J Biol Chem.

