# Supplementary material for: A P-loop Mutation in Gα Subunits Prevents Transition to the Active State: Implications for G-protein Signaling in Fungal Pathogenesis
Source: PLoS Pathog. 2012 Feb 23;8(2):e1002553. doi: 10.1371/journal.ppat.1002553 (PMC3285607; doi:10.1371/journal.ppat.1002553)
Supplement: Table S1 — Previous studies utilizing G42R mutations in fungal Gα subunits. Investigations into Gα subunit function in multiple species have included G42R point mutations. In each case, the G42R mutant was assumed to be GTPase-deficient and constitutively active. (PDF) [file ppat.1002553.s007.pdf]

Table S1. Previous studies utilizing G42R mutations in fungal Gα subunits.

**Authors**	**Year**	**Species**	**Mutation**	**Reference**
Yu, et al.	1996	*A. nidulans*	$fadA^{G42R}$	32
Hicks, et al.	1997	*A. nidulans*	$fadA^{G42R}$	33
Fang and Dean	2000	*M. grisea*	$magB^{G42R}$	26
Shimizu and Keller	2001	*A. nidulans*	$fadA^{G42R}$	34
Zuber, et al.	2002	*P. mameffei*	$gasA^{G42R}$	35
Zuber, et al.	2003	*P. marneffei*	$gasC^{G45R}$	36
Liu, et al.	2007	*M. grisea*	$magB^{G42R}$	19
Garcia-Rico, et al.	2007	*P. chrysogenum*	$pga1^{G42R}$	37
Han, et al.	2008	*A. nidulans*	$fadA^{G42R}$	38
Garcia-Rico, et al.	2008	*P. chrysogenum*	$pga1^{G42R}$	39
Garcia-Rico, et al.	2011	*P. chrysogenum*	$pga1^{G42R}$	40
