# Supplementary material for: A P-loop Mutation in Gα Subunits Prevents Transition to the Active State: Implications for G-protein Signaling in Fungal Pathogenesis
Source: PLoS Pathog. 2012 Feb 23;8(2):e1002553. doi: 10.1371/journal.ppat.1002553 (PMC3285607; doi:10.1371/journal.ppat.1002553)
Supplement: Table S2 — Data collection and refinement statistics for Gαi1(G42R) complexes. (PDF) [file ppat.1002553.s008.pdf]

Table S2. Data collection and refinement statistics for Gα_{i1}(G42R) complexes

	Gα_{i1}(G42R)·GDP/KB-752	Gα_{i1}(G42R)·GDP/RGS14 GoLoco
PDB accession	3QE0	3QI2
**Data collection**		
Space group	$P6_122$	$C222_1$
Cell dimensions *a*, *b*, *c* (Å)	106.6, 106.6, 455.1	70.0, 131.0, 203.3
Resolution (Å)	39.44 – 3.00 (3.03 – 3.00)	29.70 – 2.80 (2.94 – 2.80)
R_{merge} (%)	8.5 (52.5)	16.7 (67.3)
I/s*I*	18.5 (2.0)	8.1 (2.0)
Unique reflections	30,772 (1255)	18,462 (60)
Completeness (%)	96.7 (85.4)	78.4 (31.0)
Redundancy	3.2 (1.9)	4.4 (1.3)
Wilson B-factor (Å^2)	68.8	51.9
**Refinement**		
Resolution (Å)	39.4 – 3.0 (3.1 – 3.0)	29.7 – 2.8 (2.9 – 2.8)
No. of reflections (work/free)	29,215 / 1555 (2742 / 178)	18,462 / 952 (1729 / 88)
Cut-off (s)	1.36	0.710
R_{work} / R_{free} (%)	24.7 / 29.2 (35.1 / 40.6)	19.6 / 27.3 (28.5 / 38.4)
No. of atoms		
Protein	7470	5090
Peptide	161	515
GDP	69	46
Ions	3	16
Water	12	37
B-factors (Å^2)		
Protein	95.3	64.5
Peptide	72.4	80.4
GDP	59.8	46.1
Ions	44.4	125.9
Water	26.8	37
R.m.s. deviations		
Bond lengths (Å)	0.002	0.002
Bond angles (°)	0.546	0.463
Ramachandran		
Favored (%)	95.8	95.9
Generally Allowed (%)	4.2	4.1
Disallowed (%)	0.0	0.0
Missing residues	chain A: 30-31, 114-117, 203-205, 236-237<br>chain B: 30-31, 112-113, 348-354<br>chain C: 30-33, 113-116, 207-211, 234-239, 348-354<br>chain G: 12-16<br>chain F: 10-16	chain A: 27-29, 348-354<br>chain B: 27-33, 317, 349-354<br>chain C: 511-512, 531<br>chain D: 496-497, 510-513

Values in parentheses denote highest resolution shell
